# White-Tailed Sea Eagle (*Haliaeetus albicilla*) Die-Off Due to Infection with Highly Pathogenic Avian Influenza Virus, Subtype H5N8, in Germany

**DOI:** 10.3390/v10090478

**Published:** 2018-09-07

**Authors:** Oliver Krone, Anja Globig, Reiner Ulrich, Timm Harder, Jan Schinköthe, Christof Herrmann, Sascha Gerst, Franz J. Conraths, Martin Beer

**Affiliations:** 1 Department of Wildlife Diseases, Leibniz Institute for Zoo and Wildlife Research, 10315 Berlin, Germany; 2 Friedrich-Loeffler-Institut, Federal Research Institute for Animal Health, 17493 Greifswald-Insel Riems, Germany; Anja.Globig@fli.de (A.G.); Reiner.Ulrich@fli.de (R.U.); Timm.Harder@fli.de (T.H.); jan.schinkoethe@fli.de (J.S.); Franz.Conraths@fli.de (F.J.C.); Martin.Beer@fli.de (M.B.); 3 Agency for Environment, Nature Conservation, and Geology Mecklenburg-Western Pomerania, 18273 Güstrow, Germany; Christof.Herrmann@lung.mv-regierung.de; 4 Department of Diagnostic Investigation of Epizootics (LALLF), State Office for Agriculture, Food Safety, and Fishery, Mecklenburg-Western Pomerania, 18059 Rostock, Germany; sascha.gerst@lallf.mvnet.de

**Keywords:** HPAIV H5N8 clade 2.3.4.4b, neurological symptoms, fatal infection, white-tailed sea eagle

## Abstract

In contrast to previous incursions of highly pathogenic avian influenza (HPAIV) H5 viruses, H5N8 clade 2.3.4.4b viruses caused numerous cases of lethal infections in white-tailed sea eagles (*Haliaeetus albicilla*) affecting mainly young eagles (younger than five years of age) in Germany during winter 2016/2017. Until April 2017, 17 HPAIV H5N8-positive white-tailed sea eagles had been detected (three found alive and 14 carcasses) by real-time RT-PCR and partial nucleotide sequence analyses. Severe neurological clinical signs were noticed which were corroborated by immunohistopathology revealing mild to moderate, oligo- to multifocal necrotizing virus-induced polioencephalitis. Lethal lead (Pb) concentrations, a main factor of mortality in sea eagles in previous years, could be ruled out by atomic absorption spectrometry. HPAIV H5 clade 2.3.4.4b reportedly is the first highly pathogenic influenza virus known to induce fatal disease in European white-tailed see eagles. This virus strain may become a new health threat to a highly protected species across its distribution range in Eurasia. Positive cloacal swabs suggest that eagles can spread the virus with their faeces.

Incursions into Germany of highly pathogenic avian influenza virus (HPAIV) of subtype H5N8, clade 2.3.4.4, have been observed twice. In 2014/2015, a small number of cases in wild birds and outbreaks in poultry and a zoo were caused by HPAIV H5N8 clade 2.3.4.4a [1,2], and viruses of the same clade were also detected in apparently healthy wild birds. In contrast, the incursion in the same regions in 2016/2017 of related HPAIV H5N8 clade 2.3.4.4b caused severe clinical signs and significant mortality in a large number of infected wild birds. At Lake Plön in northern Germany, 94% (*n* = 55) and at Lake Constance in southwest Germany, 89% (*n* = 237) of the tufted ducks (*Aythya fuligula*) found dead tested positive for HPAIV H5N8 clade 2.3.4.4b [3,4]. From 9 to 21 November 2016, an obvious die-off with about 330 dead birds, among them approximately 150 greater scaups *Aythya marila*), 75 European herring gulls (*Larus argentatus*) and 25 great black-backed gulls (*Larus marinus*) [5], had been recorded on the shores of the islands of Ruden and Greifswalder Oie north of the isle of Usedom in the German part of the Baltic Sea, Mecklenburg-Western Pomerania. A portion of these birds were subsequently sampled and tested positive for HPAIV H5N8 by real-time RT-qPCR at the Germany National Reference Laboratory for Avian Influenza [4,6,7]. These events marked the onset of an unprecedented HPAIV epizootic affecting both wild birds and poultry throughout Europe.

The above-mentioned region and adjacent coastal waters along the German Baltic Sea coast represent one of the core breeding areas of white-tailed sea eagles (*Haliaeetus albicilla,* WTSE) in Germany. The first H5N8 positive white-tailed sea eagle was found in Schleswig-Holstein, northern Germany, on 15 November 2016. 

The WTSE is a strictly protected bird species breeding in Germany. It is mainly found in the eastern and northern parts of the country. In the core range, i.e., in the Federal States of Mecklenburg-Western Pomerania, Brandenburg and Schleswig-Holstein, 314, 143 and 88 breeding pairs were recorded in 2016 [8,9,10]. While adult breeding WTSE are more or less sedentary, some eagles migrate from Fennoscandian countries to the shallow lagoons of the southern Baltic Sea in Germany to spend their winter.

The increased alertness during the HPAIV epizootic of rangers of protected areas, hunters, and ornithologists for dead and diseased birds led to the notification of 17 WTSE suspect cases to local veterinary authorities. Diseased but living WTSE tested positive for HPAIV were euthanized according to legal animal disease control regulations to prevent potential further spread of the virus. Swab samples were taken from the oropharynx and the cloaca and sent to State Veterinary Laboratories for rapid diagnosis by reverse transcriptase quantitative polymerase chain reaction (RT-qPCR). For identifying the influenza A virus, we used the M gene, whereas, for the H5 subtype, we used gene-specific RNA. Diagnosis of positive cases was confirmed and extended at the German National Reference Laboratory for Avian Influenza (NRL AI) at the Friedrich-Loeffler-Institut (FLI), Isle of Riems: Submitted swab material and/or tissue specimens obtained from necropsies were used for RNA extraction. Sub- and pathotyping of confirmed H5-positive cases was carried out as already described [4]. In all cases, partial sequencing of the hemagglutinin (HA) gene confirmed the highly pathogenic phenotype (H5HP) revealing a polybasic HA cleavage site sequence of REKRRKR*G. In addition, full-length HA sequences were established from oropharyngeal swab samples of three WTSE showing high viral loads by RT-qPCR. Maximum likelihood phylogenetic analysis IQ Tree [11] of these sequences confirmed that they were deeply embedded in cluster 2.3.4.4b of the goose/Guangdong lineage of HPAI H5 viruses, and closely related to corresponding viruses obtained from other wild birds and poultry of northern and central Europe at the same time (Figure 1). Sequences are accessible from GISAID’s EpiFlu database under accession numbers EPI_ISL_320545, EPI_ISL_320546, and EPI_ISL_320574.

Necropsies were conducted under BSL-3 conditions at the FLI. For immunohistochemistry, the avidin-biotin-complex method and polyclonal rabbit anti- influenza A FPV/Rostock/34-virus-nucleoprotein antiserum was used to detect influenza A virus antigen [12]. Lead (Pb) concentration were measured in liver and kidney tissues of WTSE using the graphite furnace atomic absorption spectrometer (AAS), as previously described [13]. 

In Germany, 17 HPAIV H5N8-positive WTSE (three alive and 14 carcasses) had been detected until 3 April 2017. The last WTSE was found in early April in Hamburg (Figure 2 and Table 1).

Infected birds found alive (*n* = 3) demonstrated mild to severe neurological symptoms including torticollis, opisthotonus, limber neck, ataxia and movement in circles (Figure 3). Symptoms aggravated when the birds were stressed, e.g., by handling. Perception and reaction seemed to be normal. In contrast to symptoms seen in lead-poisoned eagles, such as depression, weakness, respiratory and coordination problems, birds infected with HPAIV were alert and overexcited. Eleven eagles examined were juvenile or immature, two were subadult and three were adult (Table 1). Necropsy showed that gross lesions characteristic for HPAI were scarce or absent. However, histopathological and immunohistological investigations revealed oligo- to multifocally necrotizing lesions in the cerebrum, cerebellum and brain stem. Immunohistological and virological investigations confirmed influenza A virus nucleoprotein antigen, and/or H5N8-specific RNA in organ samples (Figure 4). Lead (Pb) values of liver and kidney tissue revealed values within the background level of <1 ppm ruling out lead-poisoning in all cases.

Reportedly, under normal conditions, the most important mortality factor for WTSE during the winter months is lead poisoning, the second most frequent cause is collisions with trains [13,14]. During the HPAIV H5N1 clade 2.2 epidemic among wild birds in the same area in 2006, a considerable number of WTSE were found dead (*n* = 19), most of them located in the epicentre of that outbreak, the Isle of Rügen (Mecklenburg Western-Pomerania). Samples of these WTSE, in contrast to those of the current outbreak, had always tested negative for AIV [14]. Obviously, species-specific differences regarding the susceptibility and vulnerability for HPAIV infection exist among birds of prey, as peregrine falcons (*Falco peregrinus*) and common buzzards (*Buteo buteo*) died due to infections with HPAIV H5N1 in 2006 [15] and in 2016/2017 [16]. However, it cannot be ruled out that also virus strain-specific differences regarding the susceptibility and vulnerability for HPAIV infection exist and that viruses of clade 2.3.4.4b were more virulent than those of clade 2.2 in 2006/2007. It is unknown whether 2.3.4.4b HPAIV infections in WTSE are invariably lethal or if eagles can overcome the disease and acquire immunity. In addition, hooded vultures (*Necrosyrtes monachus*) and a Eurasian sparrowhawk (*Accipter nisus*) succumbed to HPAIV H5N1 infection in Western Africa in 2006 [17]. In Europe, there were no reports of HPAIV-infected WTSE until 2016. Since the fall of 2016, the epidemic of HPAIV H5 clade 2.3.4.4b induced fatal disease in WTSE in the northern part of Europe.

WTSE are facultative scavengers, feeding on carrion, especially during wintertime. If available, waterfowl is the main prey in autumn and winter [18]. Obviously, diseased and handicapped waterfowl are an attractive prey for the eagles. In any case, a top predator such as the WTSE may become repeatedly exposed to a variety of pathogens including AIV. Thus, individual birds may acquire immunity against such pathogens. The majority (two-thirds) of the affected WTSE during the current epizootic were juvenile birds, including those found alive (Table 1). We therefore conclude that juvenile and immature WTSE found infected were immunologically naïve to the virus. Older and, presumably immunologically “experienced” eagles, might have been cross-protected by heterologous immunity derived from previous avian influenza A infections. Only three eagles found were adult (older than four years of age).

Regarding the population integrity of the WTSE, a recovering and highly protected species, the relevance of the HPAIV-related death toll remains unclear. Fatal infections of WTSE with HPAIV H5N8 were recently reported around the Baltic Sea from Finland [19] Denmark, and Sweden [20]. In January 2018, a juvenile WTSE was reported to be infected with HPAIV H5N6 clade 2.3.4.4b in Ireland [21].

Generally, infected WTSE act as sentinel birds to indicate HPAIV H5Nx clade 2.3.4.4b infections in water birds in their hunting areas. Since young WTSE are roaming through their range during dispersal up to their first four to five years of life, they gather where food is abundant. HPAIV H5Nx of clade 2.3.4.4b may become a new threat to a highly protected wild bird species such as the WTSE across its distribution range in Eurasia when the virus is circulating in wild water bird populations.

## Figures and Tables

**Figure 1:**
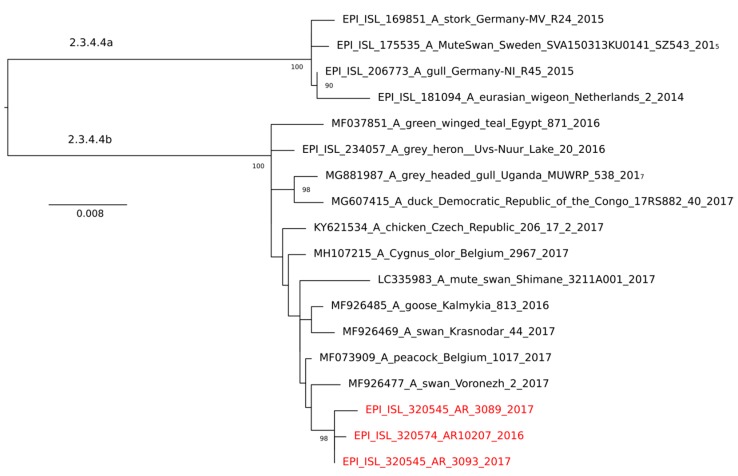
Phylogenetic analysis (maximum likelihood, IQ Tree [8]) of the full-length HA H5 open reading frame of white-tailed sea eagle (WTSE) HPAI H5 viruses (red). Significant bootstrap values supporting the distinct clades are indicated at the roots of the trees. Sequences of WTSE origin cluster in clade 2.3.4.4b of the goose/Guangdong lineage of HPAI H5 viruses and are closely related to corresponding viruses obtained from other wild birds and poultry of northern and central Europe at the same time.

**Figure 2:**
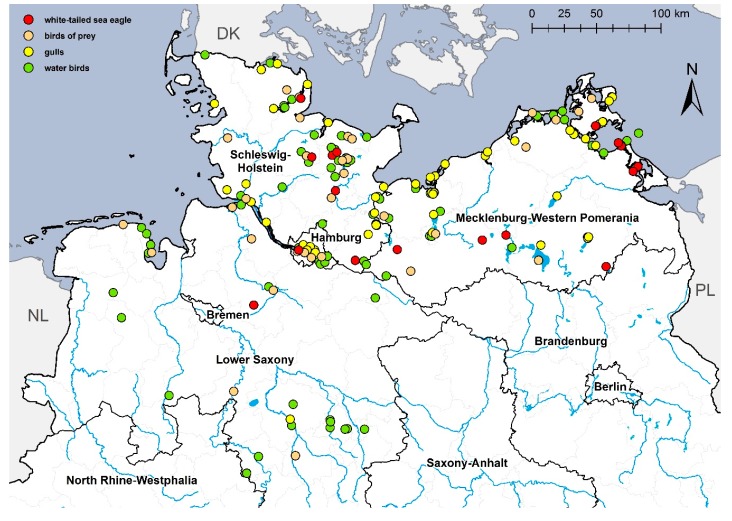
Cases of HPAIV H5N8 detected in wild birds in the federal states of Germany Lower Saxony, Schleswig-Holstein and Mecklenburg-Western Pomerania. Water birds (green dot) comprise ducks, geese, swans, herons, coots, cormorants. Birds of prey (orange dot) comprise buzzards, falcons and one owl; gulls are shown by a yellow dot; and white-tailed sea eagles are shown by a red dot.

**Figure 3:**
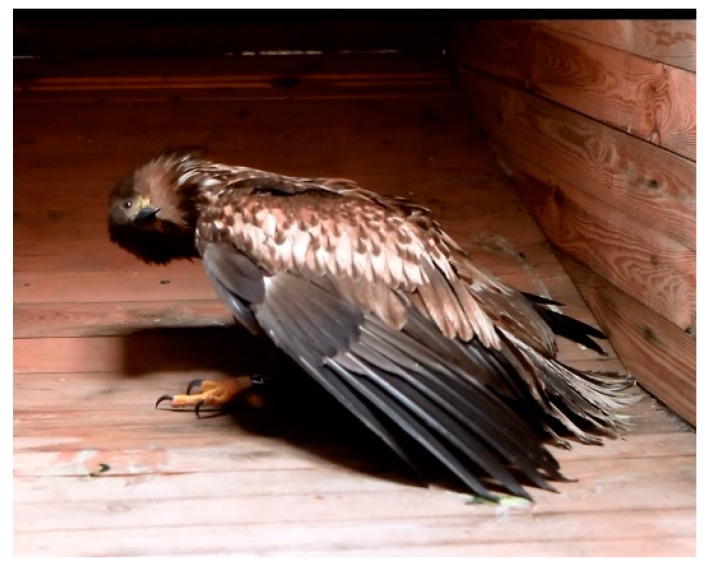
Juvenile, male white-tailed sea eagle tested positive for HPAIV H5N8 found displaying torticollis and coordination problems. The wings are dropped and the eagle is crouching on its intertarsal joints.

**Figure 4:**
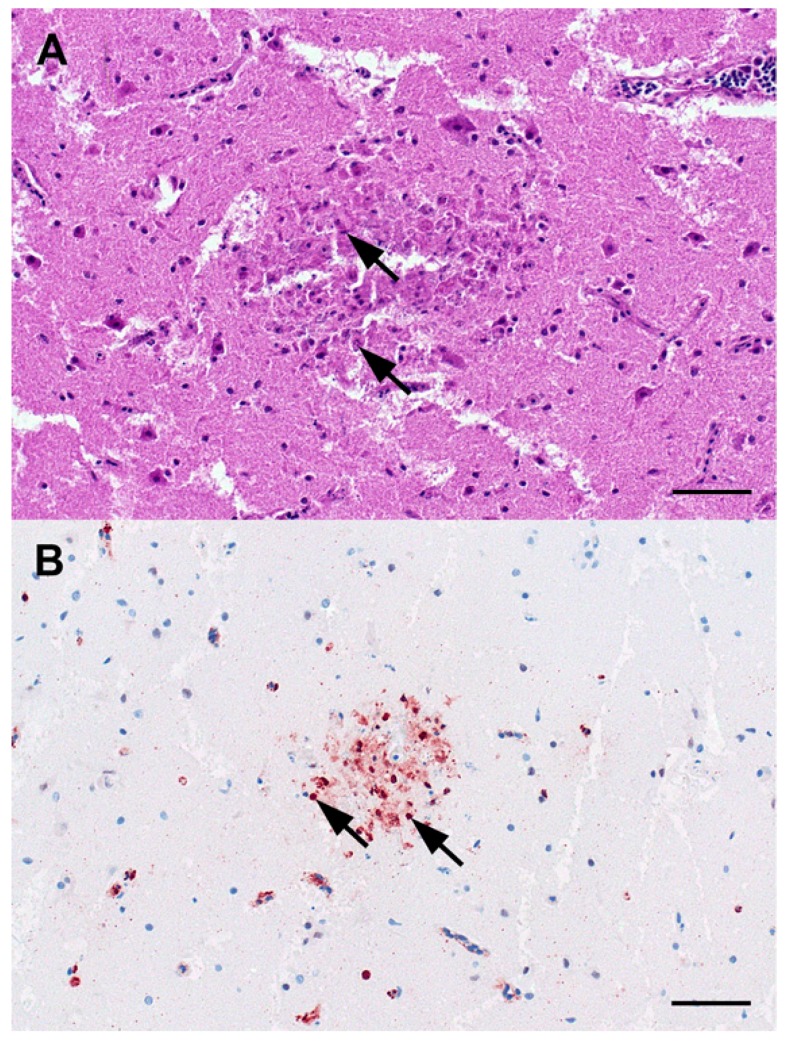
Light microscopic findings in the cerebrum of white-tailed sea eagles. (**A**) Mild, acute, oligo- or multifocal, necrotizing polioencephalitis characterized by condensed, hypereosinophilic neuroglial cells with pyknotic and karyorrhectic nuclei (arrows) represents a typical lesion. Haematoxylin–eosin; Bar = 50 µm. (**B**) Immunohistochemistry confirmed the presence of influenza A virus nucleoprotein antigen within neuroglial nuclei (arrows) within the lesions. Polyclonal rabbit anti-influenza A FPV/Rostock/34-virus-nucleoprotein antiserum; avidin-biotin-complex method; 3-amino-9-ethyl-carbazol chromogen (red-brown); haematoxylin counterstain (blue); Bar = 50 µm.

**Table 1:** White-tailed sea eagles infected with HPAIV H5N8 infection in Germany between November 2016 and April 2017 (MWP, Mecklenburg-Western Pomerania; LS, Lower Saxony; SH, Schleswig-Holstein; HH, Hamburg).

Date Eagles Found	Age	Sex	Federal State	H5	N8	HPH5
15 November 2016	Subadult	n.d.	SH	+	+	+
21 November 2016	Juvenile	female	MWP	++	++	++
27 November 2016	Adult	male	SH	+++	+++	+++
28 November 2016	Juvenile	female	SH	+++	+++	+
28 November 2016	Juvenile *	male	MWP	+	+	+
1 December 2016	Juvenile	n.d.	LS	+++	+++	+++
2 December 2016	Juvenile	male	MWP	+++	+++	+++
3 December 2016	Immature	female	SH	++++	++++	++++
3 December 2016	Immature	female	SH	++++	++++	++++
5 December 2016	Juvenile	male	MWP	++	+++	+++
10 December 2016	Immature	male	MWP	++++	++++	++++
29 December 2016	Juvenile	male	MWP	++	+++	++
29 December 2016	Adult	female	MWP	+++	+++	+++
29 December 2016	Juvenile *	male	MWP	+	+	++
9 January 2017	Juvenile	female	MWP	+	+	+
29 January 2017	Juvenile *	male	MWP	++	++	++
3 April 2017	Adult	female	HH	++	++	++

Age classes are defined as juvenile eagles up to one year, immature eagle between two and three years, subadult eagle in their fourth year and adult eagles older than five years of age; sex was not determined (n.d.) in two cases; results RT-qPCR (TC: threshold cycle): ++++ = TC < 20; +++ = TC 20–24; ++ = 25–29; + = 30–35; (+) = 36–39; asterisk indicates eagles found alive, but have been euthanized.
